# Molecular characterisation of *Staphylococcus aureus* in school-age children in Guangzhou: associations among agr types, virulence genes, sequence types, and antibiotic resistant phenotypes

**DOI:** 10.1186/s12866-023-03126-y

**Published:** 2023-11-29

**Authors:** Hao Cai, Xueying Li, Chao Zhang, Huamin Zhong, Yongqiang Xie, Lianfen Huang, Baidu Zhang, Yan Long, Zhenwen Zhou, Bingshao Liang

**Affiliations:** 1 Clinical Laboratory, Guangzhou Women and Children’s Medical Center, Guangzhou Medical University, Guangzhou, People’s Republic of China; 2 Clinical Laboratory, Longgang District Maternity and Child Healthcare Hospital, Shenzhen, People’s Republic of China; 3 National Clinical Laboratory on Tuberculosis, Beijing Key Laboratory for Drug-Resistant Tuberculosis Research, Beijing Chest Hospital, Capital Medical University, Beijing Tuberculosis and Thoracic Tumor Institute, Beijing, People’s Republic of China

**Keywords:** Agr typing, Virulence factors, Multi-locus sequence typing, Antibiotic resistance, *Staphylococcus aureus*

## Abstract

**Background:**

*Staphylococcus aureus*, one of the most prevalent opportunistic pathogens, mainly colonizes the nasal cavity and is a risk factor for severe infections. Virulence factors and accessory gene regulator (agr) are key to the severity and diversity of staphylococcal infection. In this study, we aimed to characterise *S. aureus* agr-types and virulence genes and correlated them with genetic background and antibiotic-resistant phenotypes.

**Results:**

Agr types were identified in 704 isolates (98.5%), with only 11 isolates were negative for agr type. Most of our isolates were classified as agr type I, followed by types III, II and IV. The enterotoxin c gene (*sec*) was detected in 48.6% of isolates, showing the highest prevalence among the five enterotoxin genes detected. The positivity rates for the *lukS/F-PV* and *tsst* genes were 4% and 2.2%, respectively, while neither *sed* nor *SasX* were detected. ST45, ST59, ST338, ST188, ST6, ST7, ST22, ST25, ST398, and ST944 belonged to agr I group, while ST5 and ST15 belonged to agr II group. ST30 and ST1 were classified into agr III group, and ST121 was assigned into agr IV group. The *tsst* gene was found exclusively within agr I and III types belonging to ST7 and ST30 isolates, while the *lukS/F-PV* was predominantly carried by agr I type isolates primarily within CC59 and CC22 clones. Among the methicillin-resistant *S. aureus* (MRSA) isolates, 89.7% belonged to agr I group, and 97.8% of rifampicin-resistant or intermediate isolates were assigned to agr I group. MRSA isolates harboured more tested virulence genes compared to methicillin-susceptible *S. aureus* isolates.

**Conclusions:**

We characterized the distributions of agr types and eight major virulence genes of 715 *S. aureus* isolates, and our findings revealed clear associations between agr types and STs, as well as virulence genes, and drug resistant phenotypes.

## Background

*Staphylococcus aureus* is an opportunistic pathogen that colonizes approximately 30% of the human population's nares, which is one of the most important risk factors for the development of endogenous infections [1–3]. Meanwhile, this pathogen does not only causing pneumonia, but also for sepsis and infective endocarditis with high morbidity and mortality [4, 5]. In China, infections caused by methicillin-resistant *S. aureus* (MRSA) were significantly associated with an increased clinical and economic burdens, rendering these infections more difficult to treat [6]. Notably, ST59 and ST45 emerged as two predominant clones in community-acquired MRSA infections among paediatric patients in China, with the latter being associated with respiratory tract infections [7, 8].

The pathogenicity of *S. aureus* is highly connected with its virulence factors, which were primarily regulated by the accessory gene regulator (Agr) quorum-sensing system. The agr global transcriptional regulator play a critical role in the colonization, infection and biofilm formation of *S. aureus* [9, 10]. The agr locus consists of two adjacent transcripts, RNAII and RNAIII, which are controlled by two distinct promoters. RNAII could translate into four essential proteins (agrBDCA), while the RNAIII is the small yet effective component regulating the expression of many important virulence factors. The agr locus could be divided into four groups according to sequence diversity *of agrB, agrD, and agrC* [10–12]. It is reported that strains of different agr types often exhibit different phenotypes [11].

*S. aureus* generates a wide range of virulence factors, which play key roles in toxin-mediated diseases [13, 14]. Staphylococcal enterotoxins (SEs), a class of secretory proteins with similar structure and virulence but different antigenicity, are the primary culprits behind food poisoning cases [15, 16]. Five classic enterotoxins with confirmed emetic activity are particularly significant in food poisoning incidents [17]. Toxic shock syndrome toxin-1 (TSST-1), a bacterial superantigen secreted by *S. aureus*, can activate CD4 + T cells and produce a large number of cytokines, leading to systemic toxic responses [18]. Panton–Valentine leukocidin S/F (lukS/F-PV, composed of leukocidin S and leukocidin F subunits) is a virulence factor that disrupts the cell membranes of polymorphonuclear neutrophils. lukS/F-PV-*S.aureus* is involved in invasive infection, such as necrotizing pneumonia, and can be utilized as an epidemiological marker for invasive diseases [19]. The surface protein SasX, discovered in ST239 in the UK, contributes to MRSA outbreaks by enhancing nasal colonisation, bacterial aggregation, lung diseases, and immune evasion mechanisms [20, 21].

The distribution of *S. aureus* agr types and virulence genes in the nasal colonisation of healthy children in Guangzhou received limited attention. In this study, we thus aimed to characterise agr-types and virulence genes and examine their correlations with multi-locus sequence typing (MLST) and drug resistance patterns among *S. aureus* isolates obtained from nasal swabs of Chinese children.

## Results

### Agr genotyping

Using multiplex PCR, four agr types of 715 *S. aureus* isolates were detected. Agr I group was the most prevalent, found in 62.9% of the isolates (451/715; Table 1). Agr III, agr II, and agr IV groups were found in 20.3% (145/715), 13.8% (99/715), and 1.2% (9/715) of the isolates, respectively. Eleven isolates tested negative for the agr types. The sequences of four agr groups generated were registered in GenBank with the accession codes OP997650, OP997651, OP997652, and OP997653.

**Table 1:** Prevalence of agr types and toxin genes of 715 *S. aureus* strains

Accessory gene regulator	***n*** (%)
Agr I	451 (62.9)
Agr II	99 (13.8)
Agr III	145 (20.3)
Agr IV	9 (1.2)
untyped	11(1.8)
Virulence gene, *n* (%)	
* Sea*	161(22.5)
* Seb*	75(10.4)
* Sec*	348(48.6)
* Sed*	0
* See*	12(1.6)
* lukS/F-PV*	29(4.0)
* Tsst*	16(2.2)
* SasX*	0

### Distribution of virulence genes

As shown in Table 1, the most common enterotoxin gene was *sec* (348, 48.6%), followed by *sea* (161, 22.5%), *seb* (75, 10.4%), and *see* (12, 1.6%). The positivity rates of *lukS/F-PV* and *tsst* were 4% (*n* = 29) and 2.2% (*n* = 16), respectively. The genes *sed* and s*asX* were negative in all of the strains.

### Correlation between the agr type and MLST genotype of 17 major STs

**Fig. 1:**
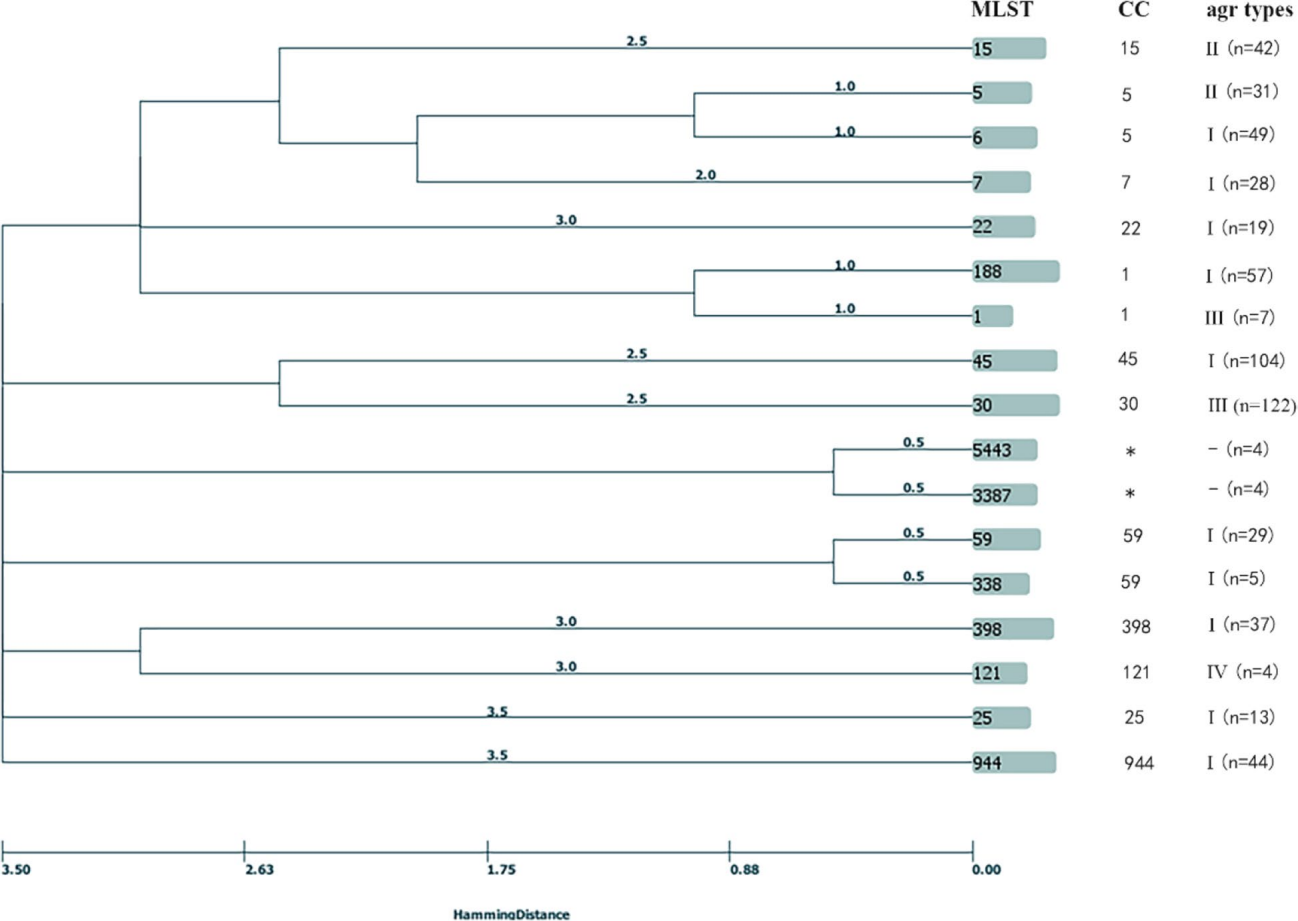
Distribution between STs and different agr groups of 599 *S. aureus* strains. MLST multi-locus sequence typing, CC clonal complex, agr accessory gene regulator, ST sequence type. * indicates agr-negative.—indicates CCs are not specified

To analyse the correlation between agr types and MLST genotypes, 17 major STs (*n* = 599) were selected, and each ST contained more than three isolates. The relationship between agr types and genotypic characteristics of the 599 *S. aureus* isolates is shown in Fig. 1. All ST6, ST7, ST22, ST25, ST45, ST59, ST188, ST338, ST398, and ST944 isolates were classified into agr I group, while ST5 and ST15 isolates belonged to agr II group. ST30 and ST1 were classified into agr III group, and ST121 was assigned into agr IV group. All ST5443 and ST3387 isolates tested negative for agr type, and their CCs were not specified in the public database. All STs and CCs could be assigned into specific agr types, except for CC1 and CC5.

### Association of agr types with virulence genes

**Fig. 2:**
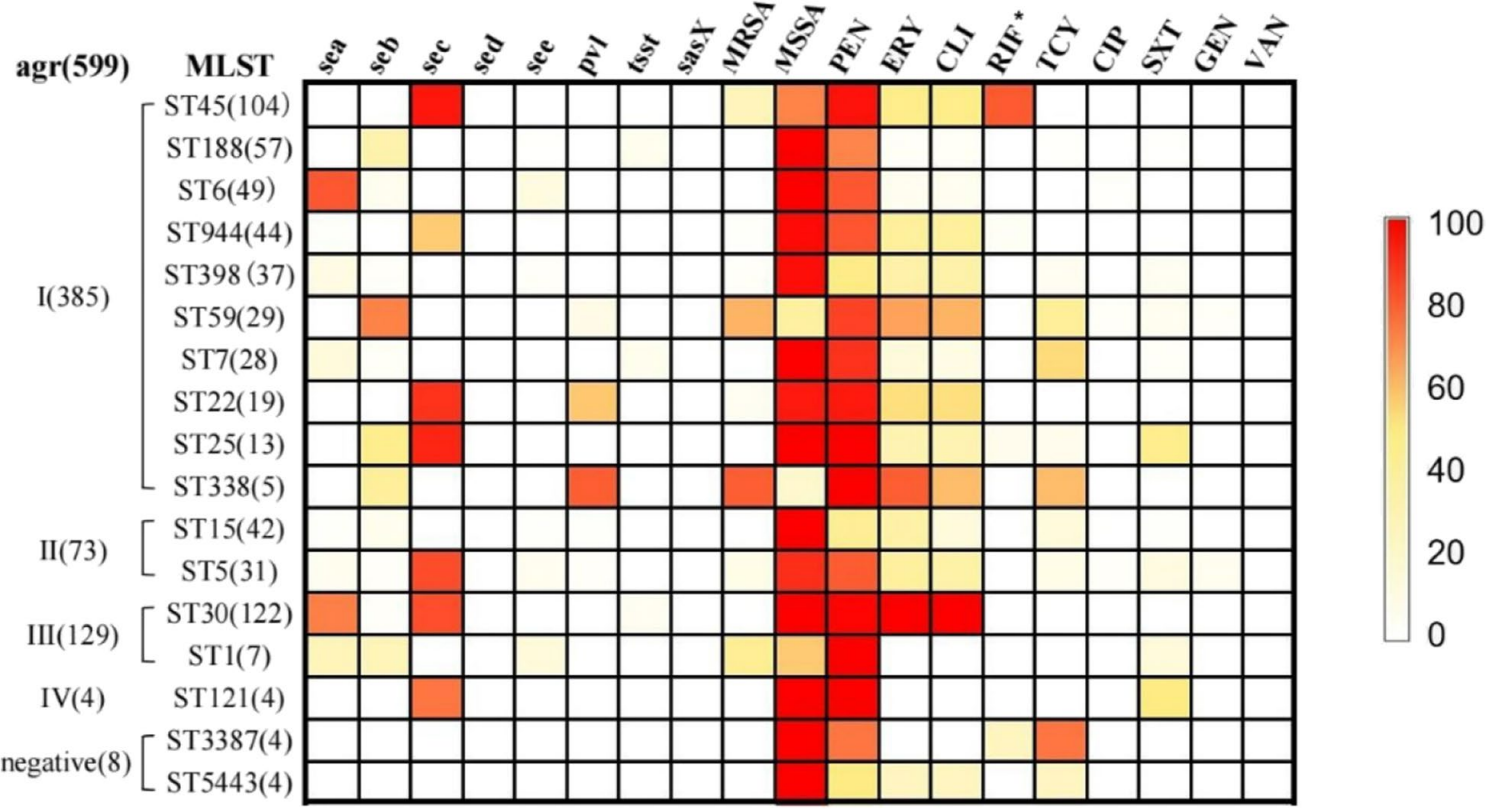
The tri-colour scale depicts the associations among *S. aureus* virulence genes, antigram, agr types, and STs. * indicates intermediate or rifampicin resistance. Antibiotics: PEN, penicillin; ERY, erythromycin; CLI, clindamycin; RIF, rifampicin; TCY, tetracycline; CIP, ciprofloxacin; SXT, sulfamethoxazole-trimethoprim; GEN, gentamicin; VAN, vancomycin

The relationships between virulence genes, antibiotic resistance and agr types are shown in Fig. 2. Of the eight virulence genes detected, four of them were strongly associated with specific agr types. The* sea* genes were more associated with agr III (59.5%) than the other three agr groups (11.3%). Nearly all isolates harbouring *seb* (66/79) and *lukS/F-PV* genes (26/29) belonged to agr I group. The *tsst* positive isolates only belonged to agr I and agr III group.

### Association of major clones with virulence genes and drug resistance

The most prevalent *sea* gene was found in agrI-ST6 (81.6%, 40/49) clone, followed by agrIII-ST30 (72.9%, 89/122). *seb* was detected in 72.4% (21/29) of agrI-ST59 clone. Approximately, 92.3% (12/13) of agrI-ST25, 83.9% (26/31) of agrII-ST5, and 75% (3/4) of agrIV-ST121 clones were found to carry *sec*. Additionally, 73.0% of agrIII-ST30 clone harboured both *sea* and *sec*, while 57.9% of agrI-ST22 clone harboured both *sec* and *lukS/F-PV* genes*.* Meanwhile, 95.2% (99/104) of agrI-ST45 clone were positive for *sec*, and 80.8% (84/104) of them were intermediate resistance or were resistant to rifampicin antibiotic; however, none of them were resistant to tetracycline (TCY) or possessed *sea, seb, sed, see, tsst*, *sasX or lukS/F-PV* genes. Eighty percent (4/5) of agrI-ST338 isolates carried *lukS/F-PV* genes. The agr negative isolates (ST5443 and ST3387) didn’t harbour any of the virulence genes. The resistance rate of ST3378 isolates to TCY was 75%, which was the same as the rate to penicillin.

The MRSA isolates predominantly belonged to agr I group, accounting for 89.7% (52/58) of the total. Meanwhile, 98.9% of rifampicin resistant or intermediate isolates were assigned to agr I group. On average, MRSA isolates harbored more tested virulence genes (107%, 75/70) than methicillin-susceptible *S. aureus* (MSSA) isolates (87.7%, 566/645) did. However, *tsst* was only found in the MSSA isolates.

## Discussion

Nasal colonisation by *S. aureus* is a notable reservoir for subsequent both local and deep seated infections. The agr system regulates the synthesis of various toxins influencing the pathogenicity and spread of *S. aureus.* Our study provides detailed insights into the presence and distribution of agr groups and specific virulence genes, and we correlated these factors with genetic profiles and antibiotic resistant patterns.

Our study identified four types of *S. aureus* agr group; agr I was the most prevalent type, accounting for 62.9% of isolates, which is similar to previous study among clinical isolates from paediatric patients in China [22]. However, the percentage of the agr I type in our study was notably lower, moreover the agr III type ranked second in this study compared to that of the third in the above article. This discrepancy may stem from our large sample size and they were isolated from 5 different schools age from 6–18 years [2], or the sources of collection and the resistant phenotype and genetic structure were different. All STs could be assigned to specific agr types, which was similar to the above in China, however, in this study, each CC was associated with a specific agr type except for CC1 and CC5. It is because CC1 and CC5 both comprised of two STs belonged to different agr types, which was accordant to the finding of a previous study using public genomes to analyse the relationship between clonal complexes and agr types, however, their results showed that CC45 contained two agr types [23]. The wide range of STs and CCs in the present study could well reflect the genetic background and their relationship with the agr groups.

Traditionally, TSS-related strains are associated with agr III [24]. However, in the present study, *tsst* was found only within agr I and III types belonging to ST7 and ST30 isolates. The high incidence of *tsst* gene carriage was reported in previous study [25], however, the *tsst*-positive agr I-ST7 isolates were seldomly reported. The *lukS/F-PV* genes was preferentially carried by agr I type isolates mainly within CC59 as previous study described [24], and in this study the *lukS/F-PV* positive ST22 isolates comprised a high proportion, which was similar to the previous studies that this high hypervirulent clone was an emerging threat especially when it’s an MRSA [26, 27]. This study showed that the detection rates of *sec* was the highest of the five classical enterotoxin genes among isolates from the nasal swabs of children, contradicting our previous findings that *seb* was the highest carriage gene [8]. These disparities could be attributed to the various sources of collected leading to different population structure of *S. aureus*.

A previous study showed that agrI-ST6 clone linked to *sea* [28], which is consistent with our findings that 81.6% of agrI-ST6 harboured this gene. Meanwhile, agrI-ST59 clone strongly associated with *seb*, and agrI-ST45 clone was correlated with *sec*. Moreover, *sea* and *sec* were specifically linked among the enterotoxins, and more than 50% of agrIII-ST30 clone simultaneously harboured *sea* and *sec*. These results indicate a link between STs and virulence genes, providing a theoretical foundation for *S. aureus* prevention and treatment.

The majority of the MRSA isolates belonged to agr I group (89.7%), and 97.8% rifampicin resistance or intermediate isolates assigned to agr I group, moreover, MRSA isolate harbored more virulence genes than MSSA did, which indicated that isolates assigned to agr I group maybe more dangerous and difficult to treat because their chances to be an MRSA carrying more virulence genes were bigger. Recently, an article showed that agr I and agr III group isolates were significantly correlated with burn severity of patients with burn wound infections [29]. In the other hand, *tsst* was exclusively found in the MSSA isolates belonging to agr I and agr III groups.

This study has certain limitations. The samples were exclusively obtained from nasal swabs, lacking representation from other body sites. It may be more comprehensive if isolates from various sources were included. Nevertheless, our study’s strength lies in its substantial sample size and multicentre samples collection, partially compensating for these limitations.

## Conclusion

In summary, we characterized the distributions of agr types and eight major virulence genes of 715 *S. aureus* isolates obtained from nasal swabs of Children in China. We have identified significant associations between agr types and STs, as well as virulence genes, and drug resistance phenotypes. These insights contribute to the knowledge necessary for the prevention and control of *S. aureus* infections*.*

## Methods

### Bacterial isolates

From February to June 2022, 715 *S. aureus* strains from the nasal swabs of children from five schools in Guangzhou were recovered. As previously mentioned, MLST and antimicrobial susceptibility tests have been performed [2].

### DNA extraction

*S. aureus* isolates were cultured, centrifuged, and resuspended in 200 μl of buffer BP, following established protocols [8]. The combination was carefully mixed after adding 5 µl of lysostaphin and placed in a water bath for digestion for 30 min. Subsequently protease and RNase were added as instruction, the mixture was incubated at 56 °C for 15 min. The solution was fully mixed with 200 μl of buffer BS-2 and equivalent amounts of anhydrous ethanol, transferred to a bacterial DNA column, and centrifuged for 1 min. The remaining steps were performed according to the instructions provided with the Accurate Biology Steady Pure Bacteria Genomic DNA Extraction Kit.

### Agr typing

Multiplex PCR was used to perform agr typing, as by Shopsin et al. described [30]. The cycling conditions were as the following: pre-denature at 95 °C for 30 s, 98 °C denaturation for 12 s, annealing at corresponding temperatures depicted at Table 2 for 28 s, extension 90 s at 72 °C for 32 cycles, and the last extension for 5 min. Two percent agarose gel electrophoresis was used to assess the PCR products, which were subjected to UV gel imaging. The size of the PCR product was determined according to the marker position.

**Table 2:** Sequences of PCR primers for Virulence genes

Genes	Sequence (5ʹ-3ʹ)	Product (bps)	TM
*agr I*	GTCACAAGTACTATAAGCTGCGAT	440	55
*agr II*	GTATTACTAATTGAAAAGTGCCATAGC	572	
*agr III*	CTGTTGAAAAAGTCAACTAAAAGCTC	406	
*agr IV*	CGATAATGCCGTAATACCCG	588	
*pan-agr*	ATGCACATGGTGCACATGC		
*sea*	GAAAAAAGTCTGAATTGCAGGGAACA	560	55
	CAAATAAATCGTAATTAACCGAAGGTTC		
*seb*	GTCAACCAGATCCTAAACCA	416	54
	ACCATCTTCAAATACCCGAA		
*sec*	AATGGCAATCCTAAACCAGA	605	55
	TCAGGCATCAAATCATACCA		
*sed*	CCAATAATAGGAGAAAATAAAAG	278	60
	ATTGGTATTTTTTTTCGTTC		
*see*	AGGTTTTTTCACAGGTCATCC	209	60
	CTTTTTTTTCTTCGGTCAATC		
*luk-PV*	GGAAACATTTATTCTGGCTATAC	502	50
	CTGGATTGAAGTTACCTCTGG		
*tsst*	ACCCCTGTTCCCTTATCATC	326	60
	TTTTCAGTATTTGTAACGCC		
*SasX*	AGAATTAGAAGTACGTCTAAATGC	615	55
	GCTGATTATGTAAATGACTCAAATG		

### Detection of virulence genes

Eight virulence genes (*sea, seb, sec, sed*, *see, tsst*, *sasX* and *lukS/F-PV*) were detected using PCR [8, 31, 32]. PCR reactions included: pre-denaturation at 95 °C for 30 s, elevated temperature denaturation for 12 s, annealing at corresponding temperatures depicted at Table 2 for 28 s, extension 90 s at 72 °C for 32 cycles, and the last extension for 5 min. Table 2 shows the target genes, primer sequences, and annealing temperatures. The positive results were randomly sent to BGI (Shenzhen, China) for sequencing. BLAST comparison was performed on the NCBI official website. The virulence gene sequences obtained in this experiment were uploaded to GenBank (http://www.ncbi.nlm.nih.gov/) under the registration numbers OP997645, OP997646, OP997647, OP997648, and OP997649.

### Phylogenetic tree construction

The MLST database (http://pubmlst.org/saureus/) was used to assign STs or clonal complexes (CCs) to all isolates. In order to represent the possible evolutionary relationship between strains, 599 *S.aureus* were clustered based on MLST data using the minimum spanning tree method in PHYLOViZ 2.0 software [22].

### Statistical analysis

GraphPad Prism 8 was used for statistical analysis. The count data were expressed as a percentage, and chi-square (χ^2^) or Fisher 's exact tests were used for comparison. *P* < 0.05 was considered statistically significant.

## Data Availability

The dataset used in this work can be accessed from the GenBank database (https://www.ncbi.nlm.nih.gov/). The GenBank registration numbers of the toxin gene sequences in this research are as follows: OP997645 (s*ea*), OP997646 (*seb*), OP997647 (*sec*), OP997648 (*tsst*), OP997649 (*lukS/F-PV*), OP997650 (*agr I*), OP997651 (*agr II*), OP997652 (*agr III*), and OP997653 (*agr IV*).

## Abbreviations

Agr: Accessory gene regulator
CCs: Clonal complexes
MRSA: Methicillin-resistant *S. aureus*
MSSA: Methicillin-susceptible *S. aureus*
MLST: Multi-locus sequence typing
lukS/F-PV: Panton–Valentine leukocidin S/F
SEs: Staphylococcal enterotoxins
TSST-1: Toxic shock syndrome toxin-1
